# Mental or Metabolic?: Misdiagnosis of Wilson Disease as Primary Psychiatric Disorder in Multiple Members of a Pakistani Family

**DOI:** 10.1192/j.eurpsy.2025.1222

**Published:** 2025-08-26

**Authors:** F. R. Bhatti, S. Liaqat

**Affiliations:** 1 Psychiatry, Pakistan Institute of Medical Sciences, Islamabad, Pakistan; 2 Psychiatry, RUSH University Medical Center, Chicago, United States

## Abstract

**Introduction:**

Wilson disease is an autosomal recessive disorder that leads to defective copper metabolism. It manifests as neurologic, hepatic and psychiatric symptoms (Benhamla et al. Encephale 2007; 33(6):924–32). Through a case series of three family members, we emphasize the varied presentation of Wilson disease, and the need for investigation of metabolic causes of such symptoms especially in families with a history of consanguinity.

**Objectives:**

We aim to underscore the importance of considering metabolic causes of psychiatric symptoms, particularly when these symptoms cluster within families.

**Methods:**

The cases are presented followed by an exploration of social context, gaps in existing literature, and suggestions for clinical practise.

**Results:**

Case 1: A 32-year-old male previously having received diagnoses of Treatment-Resistant Schizophrenia and Obsessive Compulsive Disorder over a period of 13 years presented with cognitive decline, delusions of persecution and reference, 2nd and 3rd person auditory hallucinations, preoccupation with cleaning and a significant decline in the ability to perform activities of daily living. Despite extensive psychiatric treatment, his symptoms worsened, raising suspicion for an underlying medical condition.

Case 2: A 16-year-old male, once a bright student, presented with progressive cognitive decline, poor working memory, social withdrawal, needing assistance with ADLs and sialorrhea for 2 years. These symptoms were initially misattributed to psychological stress and academic pressure, but the lack of response to treatment and worsening neurological signs of poor coordination raised suspicion for an organic cause. His eye exam was positive for Kayser Fleischer ring and his serum ceruloplasmin was below threshold level. This prompted a similar workup for his elder brother which was positive for Wilson disease.

Case 3: The mother of the aforementioned two patients, after witnessing her younger son’s decline, developed major depressive disorder with psychosis. Her symptoms started follwoing the stress of her children’s health problems and their lack of response to treatment.

Upon genetic counselling, the siblings of the first two patients revealed that they were both respectively engaged to be married to their first cousins and that their mother was adamant about these matches due to family traditions.

**Conclusions:**

These cases underscore the need for a heightened suspicion of Wilson disease and other metabolic disorders in patients who belong to regions with high rates of consanguinity and have clustering of psychiatric disorders in their family. Further studies are needed to determine the true prevalence of Wilson disease in Pakistan which may be relatively high due to the common practise of cousin marriages (Zimbrean et al. Gen Hosp Psychiatry. 2014;36(1):53–62).

**Disclosure of Interest:**

None Declared

